# Editorial: The Mechanisms Underlying the Human Minimal Self

**DOI:** 10.3389/fpsyg.2022.961480

**Published:** 2022-07-01

**Authors:** Verena Hafner, Bernhard Hommel, Ezgi Kayhan, Dongheui Lee, Markus Paulus, Stephan Verschoor

**Affiliations:** ^1^Adaptive Systems Group, Department of Computer Science, Humboldt-Universität zu Berlin, Berlin, Germany; ^2^Department of Psychology, Shandong Normal University, Jinan, China; ^3^University Hospital Carl Gustav Carus, Dresden, Germany; ^4^Department of Developmental Psychology, University of Potsdam, Potsdam, Germany; ^5^Human-Centered Assistive Robotics, Technical University of Munich, Munich, Germany; ^6^Developmental Psychology, Ludwig Maximilians-Universität München, Munich, Germany; ^7^University of Bremen, Bremen, Germany

**Keywords:** agents, self, minimal self, robotics, humanoids, cognition

The human self is a particularly colorful concept that occupies a central position in the cognitive and social sciences since their existence: it is the agent that is doing the thinking in Descartes' quest for the validity of human knowledge, the target of religious and political persuasion, the ultimate goal of personal development and therapeutic intervention, and the key factor in attributing legal and ethical responsibility. But what is the self? It is often taken as a given, or at least as a useful fiction (as in legal thinking), but rather little is known about how it works, where it comes from, and what its potential might be. Recently, there has been renewed interest in the so-called minimal self (Gallagher, 2000). According to philosophical views, the minimal self (in contrast to a narrative self or verbalized self-concept) refers to a person's phenomenal experience as an acting and perceiving individual in the here and now. In other words, it describes the pre-reflective representation that emerges from concrete sensorimotor experience. Current research has focused on, the sense of agency and body ownership experiences as two central aspects of the minimal self.

Unfortunately, the psychological basis of the minimal self is not well understood. In fact, there is no truly mechanistic approach that at least tries to capture the processes underlying the minimal self. However, important methodological developments and the availability of novel research techniques (such as virtual reality and humanoid robotics), the dramatic increase of interest in the experimental investigation of the minimal self in the recent years, and the convergence of two lines of cognitive theorizing may make the time ripe for the next major step in understanding the minimal self.

One of these lines refers to the concept of embodied cognition. There is increasing dissatisfaction with the idea that human cognition is abstract, symbolic, and entirely disembodied. This dissatisfaction has stimulated approaches that emphasize the role of people's active sensorimotor experience in creating knowledge, including knowledge about oneself. While these approaches still lack mechanistic detail (Hommel, 2016), they raise the possibility that the self is not just a given but something that emerges through experience and learning. This in turn implies that we can study and reconstruct this emergence in developmental experiments and create experimental manipulations that provide causal tests of theories by changing self-representation in predicted ways.

The other line of theorizing that provides important tools for unraveling the mechanisms underlying the self relates to ideomotor theory (Hommel, 2017). Ideomotor theory seeks to identify the mechanisms underlying goal-directed action and, given the assumed role of sensorimotor experience in creating self-representations, these action mechanisms might also contribute to understanding the mechanisms of self-creation. Unraveling these mechanisms allows researchers to reconstruct selves in artificial agents (Hafner et al., 2020), which provides a very promising testbed for empirical theories of the self. Indeed, the field of cognitive robotics becomes increasingly interested in sensorimotor learning and the re-enactment of sensorimotor experience (Vernon et al., 2015; Schillaci et al., 2016a). Internal models and mechanisms for internal simulation of sensorimotor activity have been found to be promising tools for the implementation of basic cognitive skills in artificial agents (Schillaci et al., 2016b). In particular, the ideomotor idea that decision-making is based on the anticipation of action effects plays a central role in predictive-coding approaches to both artificial agents and humans (Kilner et al., 2015).

Empirical studies of the self also strongly benefit from recent methodological developments in various fields. The study of self-development was stimulated by the availability of non-invasive brain imaging techniques (e.g., Saby and Marshall, 2012), computer-based looking time paradigms and the fine-grained analysis of eye movements and pupil size (e.g., Gredebäck et al., 2010). These methods are supposed to allow the analysis of cognitive mechanisms even in infants, yet are also under debate (Paulus, 2022). Converging ideas in developmental psychology and cognitive robotics have created a new interdisciplinary research area called “developmental robotics” (Lungarella et al., 2003; Cangelosi and Schlesinger, 2015), which seeks to implement developmental principles in behaving robots to both make robots smarter and test developmental theories “in silicio”. The availability of humanoid robots that share even basic body and sensorimotor characteristics with humans opens enormous possibilities to empirically test and improve developmental theorizing that is based on sensorimotor experience. Basic cognitive research on the self has strongly benefited from the establishment of rather simple and easy to implement paradigms, like Botvinick and Cohen's (1998) rubber hand technique, the full-body version (Petkova and Ehrsson, 2008), and the combination of the stroking technique with the visual morphing of faces (Tsakiris, 2008). Additional flexibility was provided by using virtual reality, data gloves, and advanced motion registration (Slater et al., 2010), which allows studying sensorimotor experience under very natural conditions.

The aim of the present Research Topic was to probe the level and the ambitions of current theorizing about the human minimal self. How far did we get? In particular, how far did we get in understanding the mechanistic basis of the human minimal self? How does it emerge? How is it represented? Is it stable or can it be made to disappear, as Buddhist meditation promises? These questions can hardly be answered by pointing to a particular brain area or a particular functional system of which neither the responsible codes nor the operations are specified. What is needed are theoretical assumptions that are sufficiently specific to implement them into an artificial agent and to see whether it can be made to have a self. We were thus interested in any contribution to this question, be it a theoretical comment that synthesizes available research, a review, a particular cognitive, developmental, or other kind of empirical study, a computer simulation, or a robot creating a self. Eleven contributions accepted this challenge and were selected for publication in the Research Topic.

Three Reviews summarize research highlighting the interactive roles of language and interaction, affective processing and agency, and self-other overlap and perspective taking. More specifically, Röder et al. review findings and suggested mechanisms for the grounding of language in the literature on ideomotor theory and identified computational methods that implement decision-making and verbal interaction. They outline how the available computational methods can be used to create advanced computational interaction models that integrate language grounding with body schemas and self-representations.

Kaiser et al. review the available empirical findings on how affective information modulates the experience of agency and how the sense of agency modulates the processing of affective action outcomes. They also discuss whether agency-related changes in affective processing influence the ability to enact cognitive control and action regulation during goal-directed behavior. The authors present a preliminary model that describes the interplay between sense of agency, affective processing, and action regulation. They suggest that affective processing could mediate between subjective sense of agency and the objective ability to control one's behavior.

Müsseler et al. review the available evidence on affective, cognitive, and visuo-spatial perspective taking of humans when facing or working with an avatar. They emphasize that these processes strongly depend on perceived self-avatar overlap or identification with the avatar. They discuss findings showing that when users do take the avatar's perspective, they can show spontaneous behavioral tendencies that run counter to their own.

A Mini Review by Musculus et al. addresses interoception as a crucial aspect of human minimal self in development. Extending on the embodied account of interoceptive inference, the authors present a comparative view of current theoretical frameworks explaining the link between interoception and minimal self. They propose a bi-directional link between motor and interoceptive states that jointly contribute to the formation of minimal self-early on in life. Building upon empirical findings on the development of interoception, they provide an outlook for future research addressing the knowledge gap on interoception in development.

Two Hypothesis and Theory articles address components of the minimal self. Liesner and Kunde focus on the idea of how perceptual changes (e.g., visual, auditory or proprioceptive) that are controllable by efferent activity are considered to be a part of the self. They argue that although this is highly relevant to explaining the experience of agency, sense of body ownership calls for a more nuanced distinction between proprioceptive or tactile (i.e., interoceptive) events and other controllable perceptual events.

Hommel is asking the question how people represent themselves. He proposes that they do so not any differently from how they represent other individuals, events, and objects: by binding codes representing the sensory consequences of being oneself into what he calls a *Me-File*, an event file integrating all the codes resulting from the behaving me. This approach amounts to a Human bundle-self theory of selfhood and uses recent extensions of the Theory of Event Coding (Hommel et al., 2001) for specifying the mechanisms underlying bundle-self-representation.

Two Perspective articles provide further theoretical considerations of how selves might represent themselves. Forch and Hamker discuss how two separate disciplines, namely cognitive science and cognitive robotics, approach the study of minimal self. They argue that whereas cognitive science focus on abstract models predicting and explaining empirical data obtained from humans, cognitive robotics aims at building embodied learning machines that are capable of forming a self similar to humans, which allows researchers to investigate the mechanisms underlying the emergence of the minimal self. They address the differences between human minimal self and robotics models, and provide solutions on how to create models explaining real world behavior.

Bliek et al. extend existing Bayesian models on the embodiment of physically intact limbs to amputated individuals to explain limb embodiment in structurally varying bodies. They focus on the differences in the peripersonal space, limb awareness, the use of prosthetic limbs and sensorimotor learning processes as modulators of the embodiment of artificial limbs in amputated individuals. Combining evidence from neuropsychological research with their modeling approach, they discuss implications of their approach for basic research and clinical contexts.

Three Research articles round up the Research Topic. Adam et al. examine the role of agentive experience and perceptual information on infants' processing of others' action goals. Results show that whereas 7-month-old infants did not show predictive gaze shifts, 18-month-olds did. Moreover, 11-month-olds performed predictive gaze shifts only when a salient action effect was presented. These findings point at a systematic interplay between experience-based top-down processes and cue-based bottom-up information in the development of agentive self-early on in life.

Aerdker et al. report the findings of a developmental psychological study on infant behavior concerning habituation and dishabituation in motor behavior. The study employs the experimental procedure of the habituation paradigm in a movement task to repeated action-effect situations. The experimental results provide evidence for habituation of movement generation that is specific to the direction of the movement, which supports a unified account for patterns of preferential selection based on familiarity preference. Further the authors provide a neural dynamic model that supports experimental results qualitatively and agrees with prior views regarding perceptual habituation.

Finally, Langer and Ay analyze goal-directed action from an information theoretical perspective, by measuring different information flows among the body, the brain, and the environment of an agent. They combine two theories: integrated information theory (related to measures of the amount of information integrated in the controller of the agent in order to quantify consciousness) and morphological computation (which refers to the problem from an exterior viewpoint by analyzing how the morphology of the agent and its interaction with the environment can lift the computational burden of the brain). In their experimental case study, they observe an antagonistic relationship between morphological computation and integrated information.

Overall, the Research Topic brings together different positions that contribute to current theorizing about the human minimal self. It paves the way for interdisciplinary work and will stimulate further research on how people represent themselves.

## Author Contributions

All authors listed have made a substantial, direct, and intellectual contribution to the work and approved it for publication.

## Conflict of Interest

The authors declare that the research was conducted in the absence of any commercial or financial relationships that could be construed as a potential conflict of interest.

## Publisher's Note

All claims expressed in this article are solely those of the authors and do not necessarily represent those of their affiliated organizations, or those of the publisher, the editors and the reviewers. Any product that may be evaluated in this article, or claim that may be made by its manufacturer, is not guaranteed or endorsed by the publisher.
